# Photothermal effect of albumin-modified gold nanorods diminished neuroblastoma cancer stem cells dynamic growth by modulating autophagy

**DOI:** 10.1038/s41598-022-15660-2

**Published:** 2022-07-11

**Authors:** Zahra Alizadeh Shahabad, Cigir Biray Avci, Farhad Bani, Amir Zarebkohan, Majid Sadeghizadeh, Roya Salehi, Maryam Ghafarkhani, Reza Rahbarghazi, Bakiye Goker Bagca, Neslihan Pınar Ozates

**Affiliations:** 1 Drug Applied Research Center, Tabriz University of Medical Sciences, Tabriz, Iran; 2 Department of Medical Nanotechnology, Faculty of Advanced Medical Sciences, Tabriz University of Medical Sciences, Tabriz, Iran; 3 Department of Medical Biology, Medical Faculty, Ege University, 35100 Bornova, Izmir, Turkey; 4 Department of Molecular Genetics, Faculty of Biological Sciences, Tarbiat Modares University, Tehran, Iran; 5 Stem Cell Research Center, Tabriz University of Medical Sciences, Tabriz, Iran; 6 Department of Applied Cell Sciences, Faculty of Advanced Medical Sciences, Tabriz University of Medical Sciences, Imam Reza St., Daneshgah St., Tabriz, 51666-14756 Iran

**Keywords:** Cancer, Stem cells

## Abstract

Here, we investigated the photothermal effect of gold nanorods (GNRs) on human neuroblastoma CD133^+^ cancer stem cells (CSCs) via autophagic cell death. GNRs were synthesized using Cetyltrimethylammonium bromide (CTAB), covered with bovine serum albumin (BSA). CD133^+^ CSCs were enriched from human neuroblastoma using the magnetic-activated cell sorting (MACS) technique. Cells were incubated with GNRs coated with BSA and exposed to 808-nm near-infrared laser irradiation for 8 min to yield low (43 °C), medium (46 °C), and high (49 °C) temperatures. After 24 h, the survival rate and the percent of apoptotic and necrotic CSCs were measured using MTT assay and flow cytometry. The expression of different autophagy-related genes was measured using polymerase chain reaction (PCR) array analysis. Protein levels of P62 and LC3 were detected using an enzyme-linked immunosorbent assay (ELISA). The viability of CSC was reduced in GNR-exposed cells compared to the control group (*p* < 0.05). At higher temperatures (49 °C), the percent of apoptotic CSCs, but not necrotic cells, increased compared to the lower temperatures. Levels of intracellular LC3 and P62 were reduced and increased respectively when the temperature increased to 49 °C (*p* < 0.05). These effects were non-significant at low and medium temperatures (43 and 46 °C) related to the control CSCs (*p* > 0.05). The clonogenic capacity of CSC was also inhibited after photothermal therapy (*p* < 0.05). Despite these changes, no statistically significant differences were found in terms of CSC colony number at different temperatures regardless of the presence or absence of HCQ. Based on the data, the combination of photothermal therapy with HCQ at 49 °C can significantly abort the CSC clonogenic capacity compared to the control-matched group without HCQ (*p* < 0.0001). PCR array showed photothermal modulation of CSCs led to alteration of autophagy-related genes and promotion of co-regulator of apoptosis and autophagy signaling pathways. Factors related to autophagic vacuole formation and intracellular transport were significantly induced at a temperature of 49 °C (*p* < 0.05). We also note the expression of common genes belonging to autophagy and apoptosis signaling pathways at higher temperatures. Data showed tumoricidal effects of laser-irradiated GNRs by the alteration of autophagic response and apoptosis.

## Introduction

Neuroblastoma is one of the most common brain cancers that account for about 15% of deaths in pediatric populations^1^. In recent years, various types of nanomaterials such as gold nanoparticles, magnetic nanoparticles, polymer-drug conjugates, polymeric nanoparticles, etc. have been used in cancer biology^2^. Although most nanotechnology-based cancer therapies are in the research and development stage and many attempts have been made to apply to improve cancer treatments in combination with other emerging technologies^3–5^. Photothermal therapy (PTT) is one of the nanotechnology-related hyperthermia approaches^6–10^, in which nanoparticles transform light into heat leading to cancer cell death^11,12^. PTT per se has advantages such as localized position, non-invasive, and short treatment time^12^. There are many running and completed clinical therapies regarding the application of PTT during the last decades^3,13,14^. GNRs are the most suitable nanoparticle choice for phototherapy because the shape, size, and structure of these particles are adjustable and are closely associated with optical properties^15^.

Different underlying mechanisms have been proposed in cancer cells exposed to PTT. For instance, apoptosis, necrosis, and relevant signaling pathways are activated in cancer cells upon PTT^16^. Both necrotic and apoptotic changes contribute to tumor mass atresia and shrinkage. However, the type and extent of cell death mechanisms are different from each other. During the promotion of necrotic changes, the cell membrane disrupts its integrity followed by the leakage of intracellular organelles, leading to pro-inflammatory responses. Like normal cells, in cancer cells both morphological and functional alterations can be detected after the activation of intrinsic and extrinsic apoptosis pathways. Regarding the lack of prominent inflammation in apoptotic cancer cells, thereby the activation of apoptosis has some potential benefits instead of necrosis in the context of cancer therapy. As a correlate, the development of de novo therapeutic approaches inducing cancer cell apoptosis instead of necrosis is at the center of attention^17^.

It is noteworthy to mention that the modification of PTT to cause apoptosis is promising in clinical settings^17^. The physical properties (e.g. size, shape, absorption cross-section, etc.) and passive or active targeting of nanoparticles, the intensity, and duration of the laser exposure are examples of practices that can be used to modify the cell death mechanism^18^, but the main reason for the induction of cell death through PTT is the increase of temperature by produced toxic heat under laser irradiation^19^. Although the higher increase in temperature is an efficient strategy to inhibit cancer growth and expansion the necrotic path is activated at this temperature. On the other hand, the application of a lower increase in temperatures allows the cells to provoke thermo-resistance mechanisms^18,20^.

Many studies have shown that autophagy is a necessary process for cell survival in reaction to a stressful microenvironment^21^. This phenomenon activates resistance mechanisms against different therapies such as PTT^21,22^. In this regard, previous data highlighted that the suppression of autophagy in cancer cells via pharmacological drugs and genetic profiles could increase senile cancer cells and make them more sensitive to conventional therapies^22^. Using autophagy-dependent cell survival mechanisms, cancer cells can overcome inappropriate conditions and adapt to micro-environmental changes for metastasis^23^. Dormant cells are responsible for tumor recurrence and are highly dependent on autophagy^23–25^. Cancer cells use autophagy to counteract cell death and apoptosis during dormancy. Thus, autophagy can induce dormancy^23^.

Indeed, cancer therapies induced resistance mechanisms such as autophagy in a subset of cancerous cells termed CSCs, which impairs their apoptosis and protects them against cellular stresses condition such as conventional treatments^26^. CSCs are commonly characterized by certain markers at their surface, such as CD44 and CD133^27^. Further, CSCs can reproduce and restore tumor growth and development through self-renewal properties^28^. These cells that accumulate silently away from the original tumor may lead to metastatic years after the primary tumor surgery. Meanwhile, these observations inspired researchers for more effective therapies targeting CSCs^29^.

Most previous experiments have focused on the tumoricidal effects of PTT in terms of apoptosis and necrosis of normal cancer cells rather than CSCs. Considering the dual effect of autophagy on cell dynamics and bioactivity, the close association between oncostatic effects and autophagic response after PTT has been neglected. It is suggested that deciphering the molecular identity of autophagy and its correlation with other signaling cascades in CSCs can help us to develop a suitable therapeutic approach in cancer medicine. Here, we aimed to investigate the possible oncostatic properties of PTT in the CSCs isolated from a human neuroblastoma cell line, namely SH-SY5Y cells, and address the activity of the autophagy signaling pathway. It seems that data from the current experiment could help us to assess the reaction of CSCs to PTT and the role of autophagy in cell resistance.

## Material and methods

### Materials

Hydrogen tetrachloroaurate (III) hydrate (HAuCl_4_), CTAB, silver nitrate (AgNO_3_), L-ascorbic acid, sodium borohydride (NaBH_4_), BSA, and MTT were purchased from Sigma-Aldrich (USA). Trypsin-Ethylenediaminetetraacetic acid (EDTA) and Dulbecco's Modified Eagle Medium/low glucose (DMEM/LG) were purchased from Gibco. Fetal Bovine Serum (FBS) was purchased from Hyclone® Laboratories Inc. For MACS procedure, LS Columns (Cat no: 130-042-401) and anti-human CD133 MicroBeads (Cat no: 130-097-049) were purchased from Miltenyi Biotec (Germany). For apoptosis assay, we purchased Annexin-V-fluos Staining Kit from Sigma-Aldrich. A panel of antibodies targeting autophagy such as P62 (Cat no: 5114), and LC-3 (Cat no: 3868) were purchased from Cell Signaling (USA). Purified deionized water (DW) at a resistance of 18 MΩ was used in this experiment. Methylcellulose (Cat no: M7027) was prepared from Sigma-Aldrich.

### GNRs synthesis

GNRs were synthesized by the seedless mediated method according to a previously published method^30^. Briefly, 5 ml CTAB (0.2 M) was mixed with 5 ml of 1 mM HAuCl_4_.While stirring gently for 30 min 250 µl AgNO3 (4 mM) was added to the solution. Using 37% HCl (8 µl), the acidity pH of the solution was set. Then, 70 µl of ascorbic acid (78.8 mM) was added to the solution, and stirring continued until the solution became colorless. Subsequently, ice-cold and freshly prepared NaBH4 (15 µl; 0.01 M) was added and the solution was incubated at 25 °C for 6 h. After the completion of the procedure, the solution was centrifuged at 10,000 rpm for 15 min to remove the excessive CTAB.

### Coating of GNRs with BSA

To reduce cytotoxicity and increase the stability of GNRs, we incubated the synthesized nanoparticles with BSA using two-step conjugation methods. First, GNRs (1 ml; 240 µg/ml) were diluted with DW and added slowly to 6 ml of BSA solution (5 mg/ml, pH = 7, 0.02% trisodium citrate dihydrate) during the sonication process^31^. Sonication was continued for 30 min and then the GNRs were collected using centrifugation at 10,000 rpm for 10 min. In the second step, the GNR sediment was dispersed with diluted BSA solution (0.5 mg/ml, pH = 12, 0.02% trisodium citrate dihydrate). The solution was incubated at 25 °C and stirred overnight at speed of 50 rpm. Then the particles were centrifuged and washed with deionized water.

### Characterization of GNRs

The morphology and size of GNRs and BSA-GNRs were detected using TEM (Model: LEO 906, Germany). To this end, one drop of GNR solution was placed on a copper grid and left at room temperature for 10 min to dry. The grids were visualized at the voltage of 80 kV. Other features such as zeta-potential and hydrodynamic size were measured (Zetasizer Nano ZS90; Malvern Instruments, Malvern, UK). The ultraviolet–visible spectrum of GNRs was obtained using a double beam UV–Vis spectrophotometer (UV-160, Shimadzu Corporation; Japan). Atomic absorption spectrometry was used to determine the precise concentration of GNRs in the solution of GNR and GNR-BSA. The Solutions were dissolved in aqua regia overnight and heated up to 140 °C to eliminate hydrogen chloride and nitrogen oxide until the solutions became colorless and clear. Thereafter, 2 ml solution was prepared using an aqueous solution containing 2% nitric acid and 1% hydrogen chloride, and the total gold content was calculated using an atomic absorption spectrometer.

### The photothermal effect of GNR-BSA conjugate

The prepared GNRs-BSA dispersion (30 ppm Au content) was irradiated using an 808 nm near-infrared diode laser (MDL-III-808–2.5 W, Changchun New Industries Optoelectronics Technology Co., Ltd). The irradiation procedure was performed at the intensity of 0.5, 1, and 1.5 W/cm^2^ for 8 min. The dispersion temperature was measured pre- and post-irradiation by a digital thermometer equipped with a thermocouple probe (Pyrometer Instrument Company).

### Cell culture protocol

In this study, we aimed to assess the tumoricidal effect of GNRs on the human neuroblastoma SH-SY5Y cell line. Cells were obtained from Stem Cell Research Center, Tabriz University of Medical Sciences. Cells were cultured in DMEM/LG with 10% fetal bovine serum (FBS) and 1% Penicillin–Streptomycin (Pen-Strep). Cells were plated at an initial density of 1 × 10^4^ per cm^2^ culture plate and kept at 37 °C and humidified atmosphere with 5% CO_2_. The medium was replenished every 2–3 days. SH-SY5Y cells at 70–80% confluence were passaged using 0.25% Trypsin–EDTA solution. Cells between passages 3–6 were used for the current project.

### Enrichment of CD133 + CSCs using MACS

To assess whether irradiated GNRs can exert tumoricidal effects on CSCs, we enriched CD133^+^ using MACS^32^. To this end, SH-SY5Y cells were detached and collected using a 0.25% Trypsin–EDTA solution. Cells were incubated in 1% BSA for 20 min followed by incubation with human CD133 MicroBeads at 4 °C while shaking. To separate CD133^+^, the samples were passed through LS Columns attached to the MidiMACS magnet. Following twice PBS washes, the purity of enriched CD133^+^ cells was assessed by anti-human FITC-conjugated CD133 antibody using BD FACSCalibur system and FlowJo software (Ver. 7.6.1).

### Cell survival assay

The viability of SH-SY5Y CSCs was monitored after exposure to PTT using a conventional MTT assay. For this purpose, 1 × 10^4^ SH-SY5Y CSCs were plated in each well of 96-well plates and incubated for 24 h. Cells were cultured in DMEM/LG with 1–2% FBS and randomly allocated into different groups as follows; Control, GNR (30 ppm), GNR-BSA (30 ppm Au content), Hydroxychloroquine (HCQ; 20 μM), Laser/GNR (43 °C), Laser/GNRs (46 °C), Laser/GNRs (49 °C), Laser/GNRs/HCQ (43 °C), Laser/GNRs/HCQ (46 °C), and Laser/GNRs/HCQ (49 °C). In this study, we aimed to assess the autophagic CSC death after the application of PTT. Therefore, HCQ was used as an autophagy inhibitor to assess whether the inhibition of autophagy can exacerbate the tumoricidal effect of GNRs. To this end, CD133^+^ CSCs were pretreated with 20 µM HCQ and 30 ppm of GNRs or GNRs-BSA for 4 h. After completion of incubation time, the supernatant was discarded and CSCs incubated in a fresh culture medium and irradiated by different intensities of the laser for 8 min to reach temperatures of 43, 46, and 49 °C. After 24 h, the survival rate was measured using the MTT assay. The supernatants were replaced with 5 mg/ml of MTT solution and incubated at 37 °C for 3–4 h followed by the addition of 100 µl pure dimethyl sulfoxide. The plates were shaken gently and the OD of each sample was measured at 570 nm using a microplate reader (Awareness Technology, USA). The cell viability values were expressed as % of the non-treated control group.

### Measuring the apoptosis rate by flow cytometry analysis

We also measured the rate of apoptosis in CD133^+^ CSCs after the PTT protocol. In short, 10^5^ CD133^+^ cells were seeded in each well of 24 well plates and kept for 24 h. Four hours before the PTT, cells were treated with GNR, GNR-BSA, HCQ, and GNR-BSA/HCQ. Irradiation was done using the laser at different intensities for 8 min to reach temperatures of 43, 46, and 49 °C. After 24-h incubation time, the rate of apoptosis was calculated using Annexin-V-fluos Staining Kit according to the manufacturer’s instruction using BD FACSCalibur® system and FlowJo software (ver. 7.6.1).

### PCR array analysis

The modulatory effect of PTT on autophagy signaling transduction pathways was investigated using PCR array analysis. After the completion of the treatment protocol, CD133^+^ CSCs were collected and total RNA content was extracted using Qiagen RNAeasy kit. Next, RT^2^ First Strand Kit (SABiosciences) was used for cDNA synthesis. The modulatory effect of the PTT procedure on the autophagy signaling pathway was monitored using the Human Autophagy RT^2^ Profiler PCR Arrays (PAHS-084Z, SABiosciences) profiles targeting 84 genes involved in the autophagic response. Real-time PCR reaction was done using the Light Cycler 480 System II (Roche) and results were processed using 2^−ΔΔCT^ (Light Cycler 480 quantitative software) and compared to the expression of control housekeeping genes. Web-based RT^2^-based PCR array analysis (SABiosciences) was used to represent the data as a fold change expression. Differences in expression more than twofold were accepted as the cut-off value. *p* < 0.05 was considered statistically significant. This assay was performed in triplicate.

### ELISA

To this end, the protein levels of LC3, and P62 were measured using ELISA. After completion of the PTT procedure at different received temperatures (43, 46, and 49 °C), CSCs were collected from different groups and lysed with the lysis buffer consisting of NaCl (50 mM), sodium dodecyl sulfate (SDS; 0.1% w/v), Tris–HCl (50 mM), EDTA (2 mM), and NP-40 (1% v/v). After overnight incubation, the samples were centrifuged at 14,000 rpm at 4 °C for 20 min. The levels of autophagic factors such as P62 and LC3 were measured using the ELISA method designed by our research group. 100 µl of antibody solution (1 µg/ml) was placed in each well of polystyrene 96-well plates (SPL) and plates were maintained overnight at 4 °C. Then, 1% BSA solution was added to each well and kept for 1 h at RT after the supernatant removal. Thereafter, 100 µl of different samples at a protein concentration of 10 µg/ml was added to each well. After 1 h, the wells were washed twice using Tris-buffered saline-0.1% Tween 20 (TBST) solution (each for 10 min), and 100 µl HRP-conjugated secondary antibody was poured into each well. After three-time TBST washes, 50 µl 3, 3′-Diaminobenzidine was used. The reaction was stopped using 5% sulfuric acid and ODs were determined at 450 nm using a microplate reader.

### Clonogenic (tumorsphere formation) assay

CSCs have the potential to form colonies, indicating putative self-renewal capacity and stemness feature^33^. After the completion of the PTT protocol, CSCs were collected from each group and counted. Thereafter, 1 × 10^3^ CSCs were mixed with 1 ml cell culture gel consisted of 2.5% methylcellulose, 0.1% agar, 2% FBS, 1X DMEM/LG^34^. The cell suspension was immediately transferred into each well of 6-well plates and allowed to solidify. Two weeks after seeding, the number of colonies (tumorsphere) was counted under an inverted microscope and compared to the control group.

### Statistical analysis

Data were represented as mean ± SD. Using One-Way ANOVA and Tukey post hoc analysis; we monitored statistically significant differences between the groups. *p* < 0.05 was statistically considered significant. Three sets of experiments were done in this study otherwise mentioned.

### Ethics approval

All procedures of this study was approved by the Local Ethics Committee of Tabriz University of Medical Sciences (IR.TBZMED.REC.1397.180).

## Results

### Characterization of GNRs

**Figure 1:**
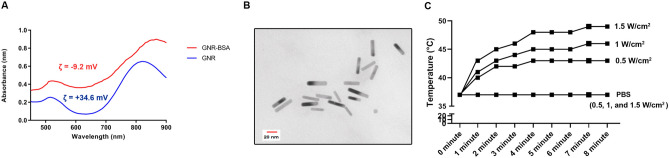
UV–Vis spectrum of GNRs and GNR-BSA (**A**); TEM imaging revealed rod-shaped gold nanoparticles reaching mean length and diameter of 32.1and 7.7 nm (**B**); Photothermal effects of GNR-BSA exposed to 808 nm near-infrared diode laser for 8 min at intensities of 0.5. 1, and 1.5 W/cm^2^ leading to ambient temperature increase (**C**).

CTAB-capped GNRs with positive surface charge were synthesized using the seedless method^30^. According to UV–Vis spectroscopy measurements, the maximum localized surface plasmon resonance (LSPR) was exhibited at 819 nm (Fig. 1A). TEM imaging revealed relatively monodispersed rod-shaped GNPs, indicating the efficiency of the current synthesis protocol (Fig. 1B). Data showed the mean length and diameter of GNRs reached approximately 32.1 ± 4.6 and 7.7 ± 1.0 nm, respectively. Besides, DLS displayed a mean hydrodynamic size of 77 ± 11.6 nm with a zeta potential value of + 34.6 ± 4.8 mV. In this study, the surface of GNRs was coated with BSA using a ligand exchange reaction to reduce the toxicity and increase the stability of particles^31^. Noteworthy, the zeta potential of BSA-GNRs was changed to -9.2 ± 1.2 mV after BSA coating and the hydrodynamic size of BSA-GNRs was slightly larger than that of CTAB-GNRs. Along with these changes, UV–Vis spectroscopy showed a redshift (~ 20 nm) in LSPR of the GNR post-BSA coating.

### Evaluating photothermal properties of GNRs-BSA

Because of unique LSPR properties, GNRs are eligible nanoparticles to generate heat when exposed to near-infrared laser^35^. To calculate the photothermal capacity, we recorded the temperature increment of BSA-GNRs (30 ppm Au content) in an aqueous solution and PBS solution as a control sample after being exposed to 808 nm laser irradiation of 0.5, 1, and 1.5 W/cm^2^. The irradiation procedure was continuously applied for 8 min and temperature changes were recorded using a digital thermometer. As shown in Fig. 1C, the solution temperature was increased by time from the start temperature of 37 °C in which maximum temperatures were obtained after 8 min in all groups except the PBS group compared to the other time points. As expected, these effects were heightened by increasing the intensity of laser intensity. We noted that the ambient temperature in the presence of BSA-GNRs increased to 43, 46, and 49 °C in 0.5, 1, and 1.5 W/cm^2^ groups. According to our data, no temperature variation was found in the PBS group after exposure to the 808 nm laser irradiation with three temperature settings. These data show that the ambient temperature of a solution containing BSA-GNRs can be adjusted by controlling the time and laser intensity.

### Irradiated GNR-BSA alone or in combination with HCQ decreased the viability of CD133^+^ CSCs

**Figure 2:**
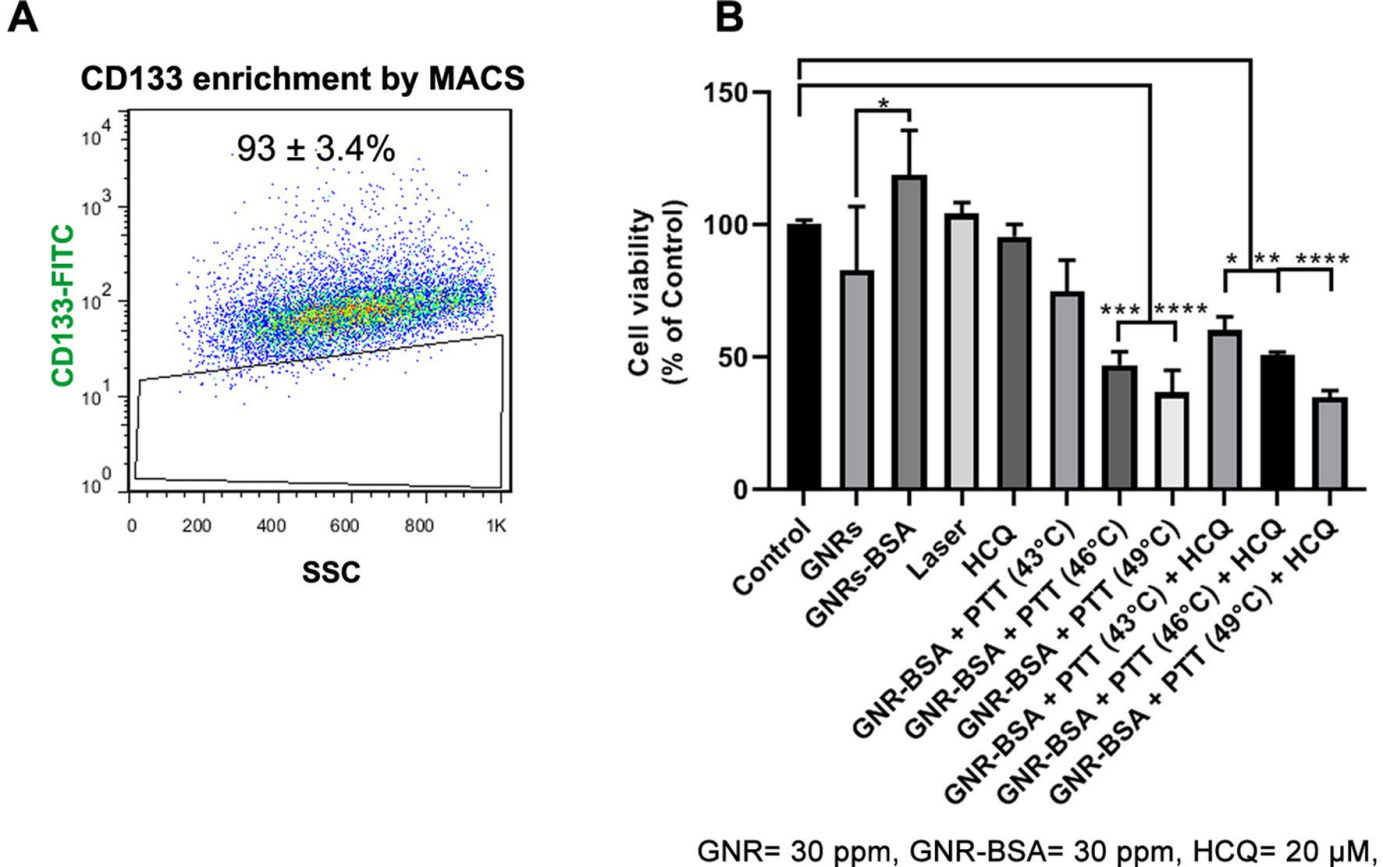
Enrichment of CD133^+^ CSCs using MACS (**A**). Over 90% of isolated cells were positive for surface marker CD133. Measuring the CSC survival rate using MTT assay (**B**). Data showed the reduction of GNRs toxicity after coating with BSA. The exposure of CSCs to low, medium, and high temperatures caused CSC viability rates compared to the control group. The combined regime of PTT and HCQ did reduce cell survival as compared to the non-treated CSCs. One-Way ANOVA and Tukey post hoc analysis. *p < 0.05; **p < 0.01; ***p < 0.001 and ****p < 0.0001.

Flow cytometry analysis revealed high-rate purity of enriched CD133^+^ CSCs after the MACS procedure. MTT assay was used to assess tumoricidal effects of GNR-BSA conjugate and laser irradiation on CD133^+^ SH-SY5Y cells after 24 h (Fig. 2A). Cells were exposed to the combined regime of GNR-BSA and laser irradiation at different intensities leading to the ambient temperature of 43, 46, and 49 °C, respectively. It was suggested that the conjugation of BSA to the GNRs reduced CSC toxicity (*p* < 0.05; Fig. 2B). GNRs were coated with the BSA to stabilize their structures and reduce toxicity. According to our data, a 4-h incubation of CD133^+^ CSCs with GNR-BSA did not alter cell survival rate compared to the control and GNR groups (*p* > 0.05). Similarly, treatment of CD133^+^ SH-SY5Y CSCs with 20 µM HCQ and the used highest laser intensity did not change the survival rate compared to the control (*p* < 0.05). The application of both GNR-BSA and laser irradiation (43, 46, and 49 °C) reduced the survival rate compared to the non-treated control group (*p* < 0.05), indicating the tumoricidal effect on CSCs. Likewise, the simultaneous addition of HCQ to GNR-BSA and PTT groups led to a significant reduction in survival rate as compared to the control group (*p* < 0.05; Fig. 2B). No significant differences were obtained in groups treated at 43, 46, and 49 °C for 8 min. Although the cytotoxicity rate was slightly more in GNR-BSA + PTT + HCQ CSCs compared to GNR-BSA + PTT matched control groups no statistically significant differences were achieved. These data demonstrate that treatment of CSCs with the combination of GNR-BSA + PTT or GNR-BSA + PTT + HCQ can diminish the survival rate.

### GNR-BSA and laser irradiation-induced apoptosis

**Figure 3:**
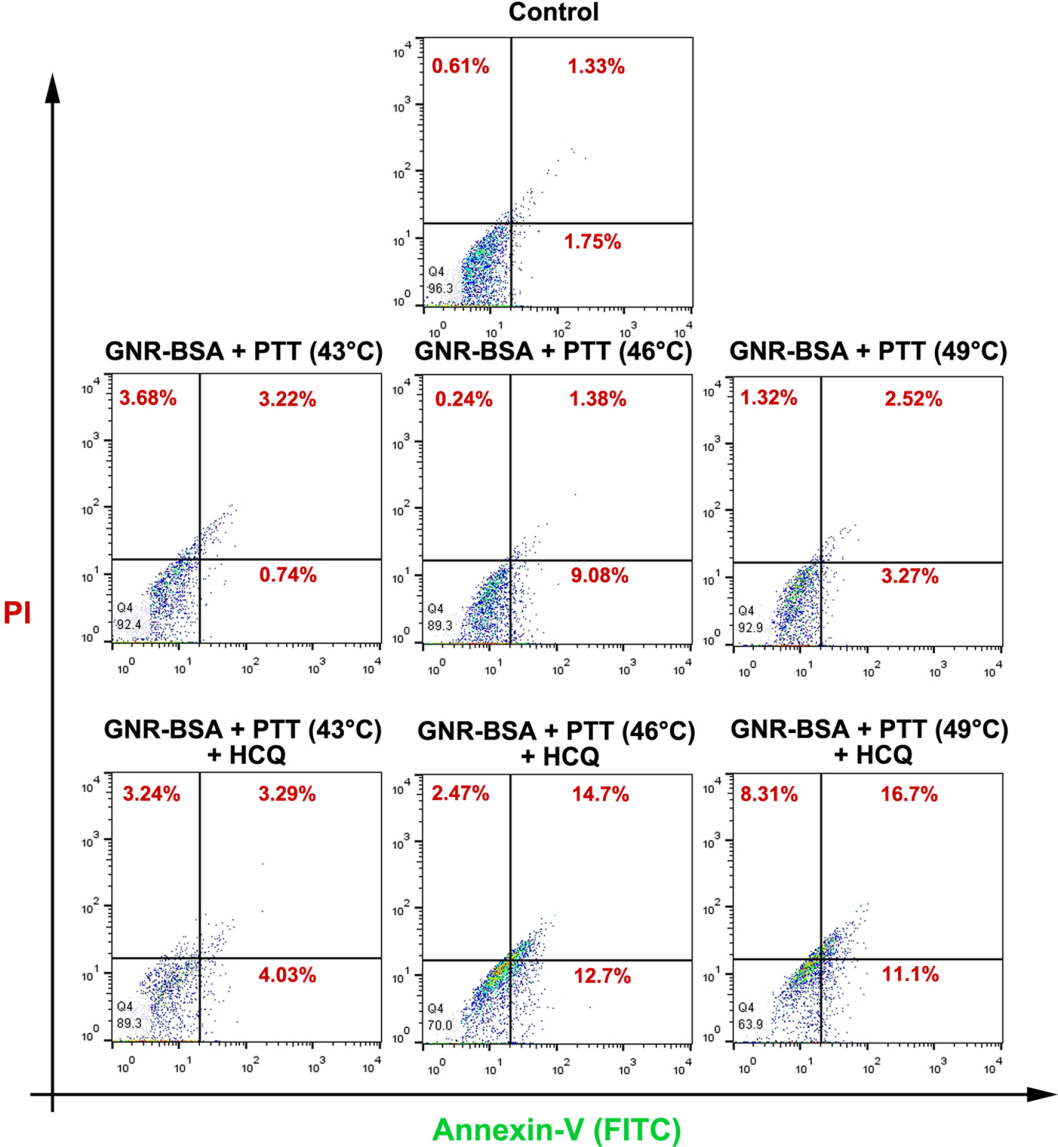
Flow cytometric analysis of apoptosis and necrosis using Annexin-V and PI staining. Data showed the induction of apoptotic CSCs by increasing temperature from low to high temperatures.

Previous experiments have shown that the cell death mechanism induced by PTT can be related to the necrotic and apoptotic changes^28^. Here, we performed flow cytometric analysis of Annexin-V and PI to calculate the percent of both apoptotic and necrotic cells (Fig. 3). The combination of GNR-BSA with laser irradiation at 43, 46, and 49 °C for 8 min increased the number of cells in early and late apoptotic changes. It was suggested that concurrent treatment with GNR-BSA, laser irradiation, and HCQ increased the values in which 8.3 and 27.8% of CSCs exhibited necrotic and apoptotic changes after exposure to 49 °C for 8 min in the presence of HCQ (Fig. 3). It seems that the fraction of apoptotic CSCs was induced by increasing the temperature from 46 to 49 °C pretreated with the HCQ for 4 h (Fig. 3). In the presence of HCQ, irradiated GNR-BSA can exert tumoricidal effects on CD133^+^ CSCs at moderate and higher temperatures (46 and 49 °C) via necrosis rather than apoptosis. These data noted that apoptotic death is probably the main CSC death mechanism after being exposed to GNR-BSA plus PTT.

### The photothermal activity of GNR-BSA induced autophagy response in CSCs

**Figure 4:**
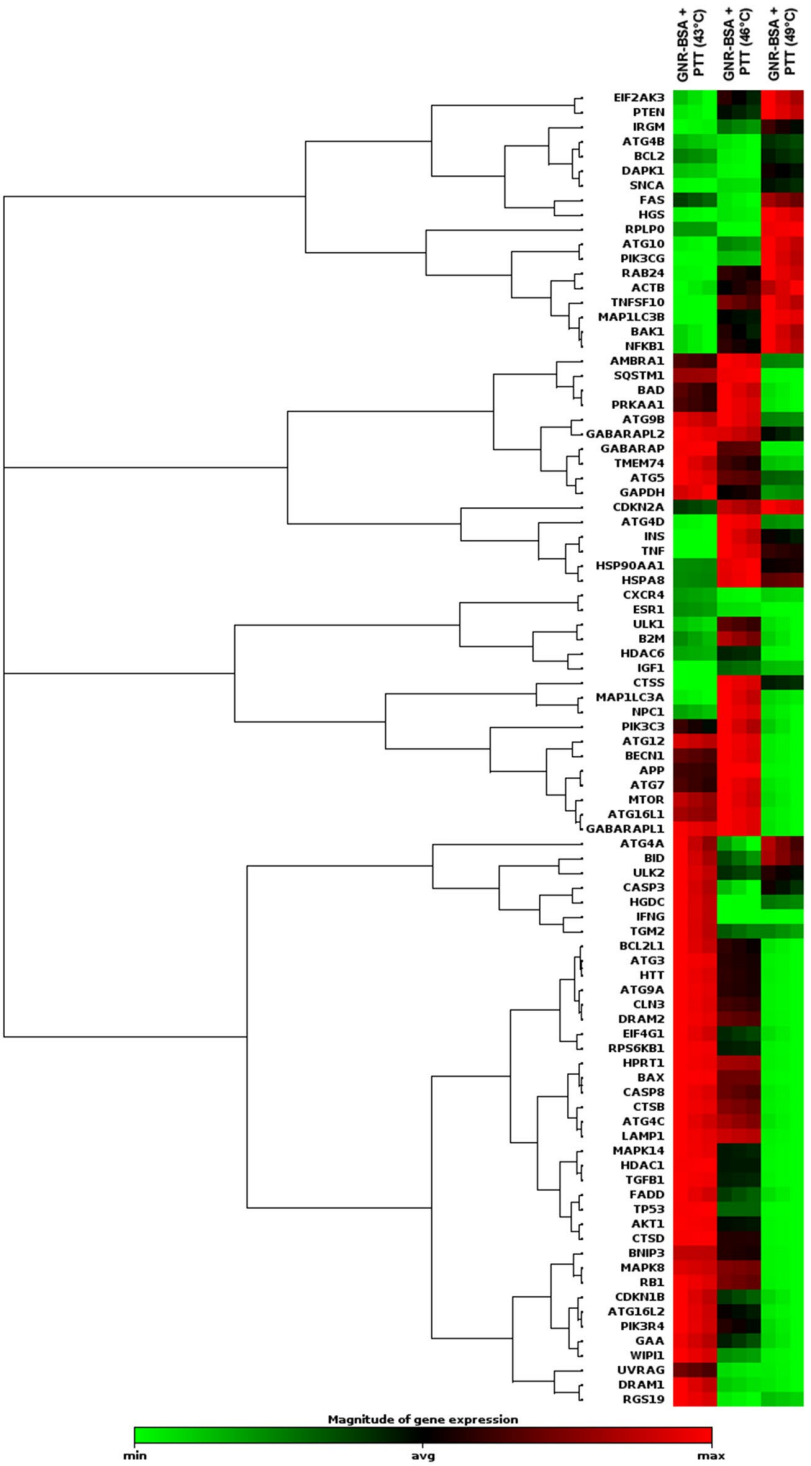
Clustergram illustration of PCR array for autophagy signaling. Over 80 genes were monitored in CSCs after 8-min exposure to 43, 46, and 49 °C.

The status of autophagy response is critical in CSCs resistance to therapeutic modalities^36^. Here, we performed PCR array analysis of the autophagy signaling pathway to assess the expression of autophagy machinery components from different signaling transduction pathways (Table 1, and Fig. 4). Based on our data, 8-min exposure of CSCs suppressed the expression of Akt1 (~ threefold) compared to the control CSCs. Besides, the expression of MAP1LC3B (~ 4.7-fold), and ATG10 (~ 3.6-fold) participating in autophagic vacuole formation and protein transport procedure was significantly induced in the group received 49 °C. The transcription of ATG9B was increased in all groups compared to the control group. Despite the induction of GABARAP at 43 and 46 °C, 8-min incubation of CSCs at 49 °C blunted the expression rate. Along with these changes, the expression of WIPI1 and ULK1 was decreased at a similar thermal value. Of note, transcription of AMBRA1 was also induced in groups with thermal stress of 43 and 46 °C. By contrast, expression of ATGL16L2 and RGS19 was also suppressed in the group treated at 49 °C. It was suggested that ATGL16L2 can act as a dominant-negative inhibitor of autophagy and compete with ATGL16L1 for interacting with ATG5, indicating suppressed autophagy response via inactivation of LC3 and proteasomal degradation^37^. In contrast, the expression of MAP1LC3A was suppressed at high ambient temperatures related to the control levels. Compared to the control CSCs, the expression of BCL-2, DRAM1, CXCR4, DAPK1, and BNIP3 was significantly inhibited after thermal stress (*p* < 0.05; Table 1, and Fig. 4). These factors act as co-regulators of autophagy and apoptosis. Despite the slight increase in the expression of ATG5, no statistically significant differences were found in GNR-BSA + PTT (49 °C) group compared to the control CSCs. Among the majority of genes participating in autophagic vacuole formation, the expression of ATG5, ATG7, ATG9A, and AMBRA1 displayed the lowest in groups that received 49 °C for 8 min, showing inhibition of vacuole formation in a temperature-dependent manner. The suppression of CLN3 in the GNR-BSA + PTT (49 °C) group revealed the problem is associated with protein cargo transport into the lysosomal system. The expression of DRAM1 and 2 was also inhibited by the increasing temperature. According to our data, the transcription level of ESR1 showed ~ 4.28, 12.38, and a 38.75-fold decrease in CSCs exposed to 43, 46, and 49 °C. The reduced expression of ubiquitination-associated genes such as HDAC6 showed an abnormal ubiquitin–proteasome system, especially at the ambient temperature of 49 °C. The inhibition of IFNG and SNCA after thermal stress showed apoptosis induction by the inhibition of cell cycle activity. IFNG is a mediator between the cell cycle dynamics, autophagy response, and apoptosis. Similar to IFNG, the expression of IGF1, RPS6KB1, and RB1 were also suppressed (Table 1, and Fig. 4). These data showed the cooperation of both autophagy and apoptosis to yield tumoricidal effects. The − 4.25-Fold reduction in the expression of MAPK8 showed the efficiency of our protocol in the promotion of apoptotic changes. MAPK8 can increase the tumor cell's resistance to the insulting condition^38^. Therefore, it is suggested that the suppression of this factor is a hallmark of apoptosis induction. Unlike, the PIK3CG gene was up-regulated especially at a temperature of 46 and 49 °C. This would be possibly related to the compensatory response to CSCs against thermal treatment. Of note, we found near 7101-fold induction in the expression of the TNF gene at the temperature of 46 and 49 °C, showing prominent pro-inflammatory conditions. The induction of TNFSF10 at these temperatures showed the induction of autophagy via the TNF receptor. According to our data, the induction of both HSP90AA1, and HSPA8 genes under low, medium, and high temperatures showed the activation of chaperon-mediated autophagy.

**Table 1:**
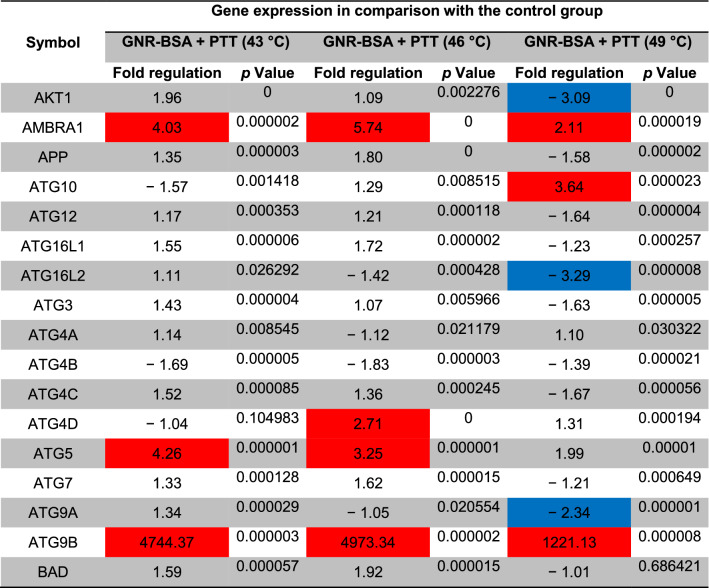

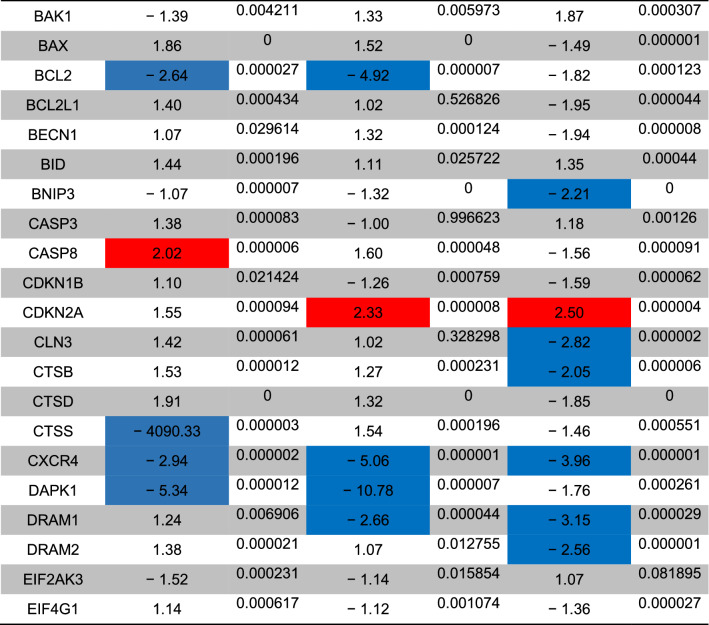

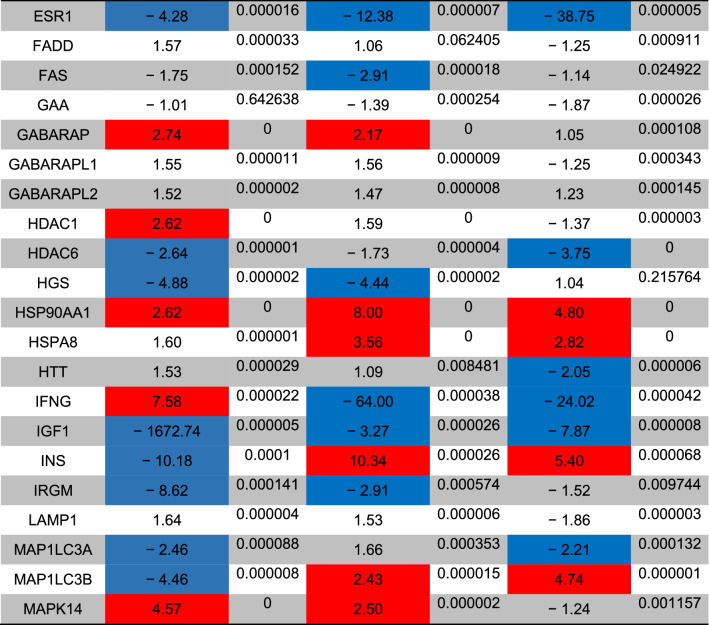

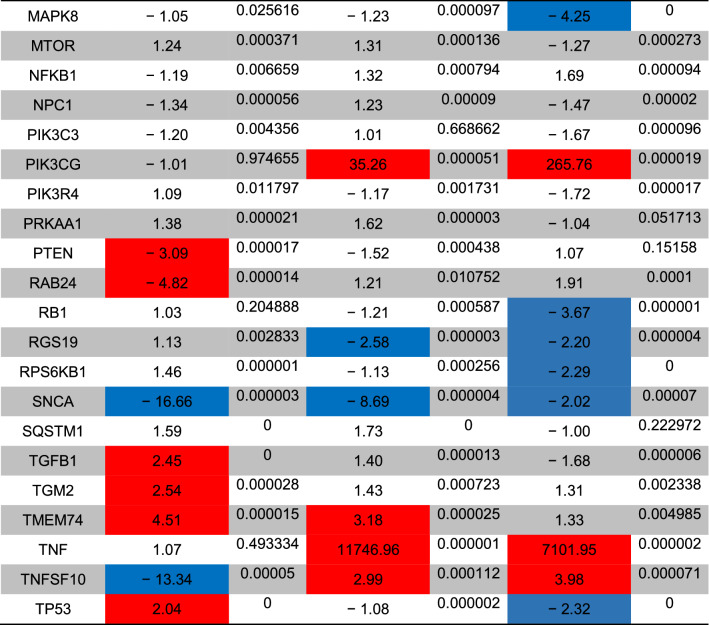

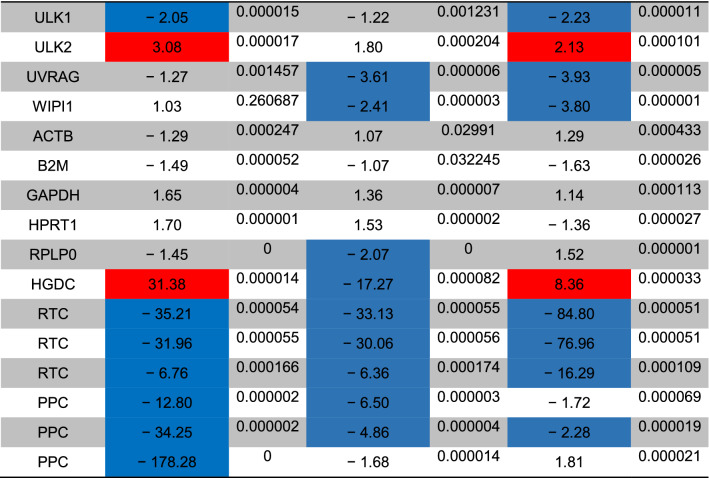
Monitoring the expression of autophagy-related genes using PCR array analysis.

The *p* values are calculated based on a Student’s t-test of the replicate 2^(−∆∆Ct)^ values for each gene in the control group and treatment groups, and *p* values less than 0.05 are indicated in red for genes with twofold increase and blue for genes with -twofold decrease (n = 3).

### The autophagic response was aborted in CSCs exposed to the PTT procedure

**Figure 5:**
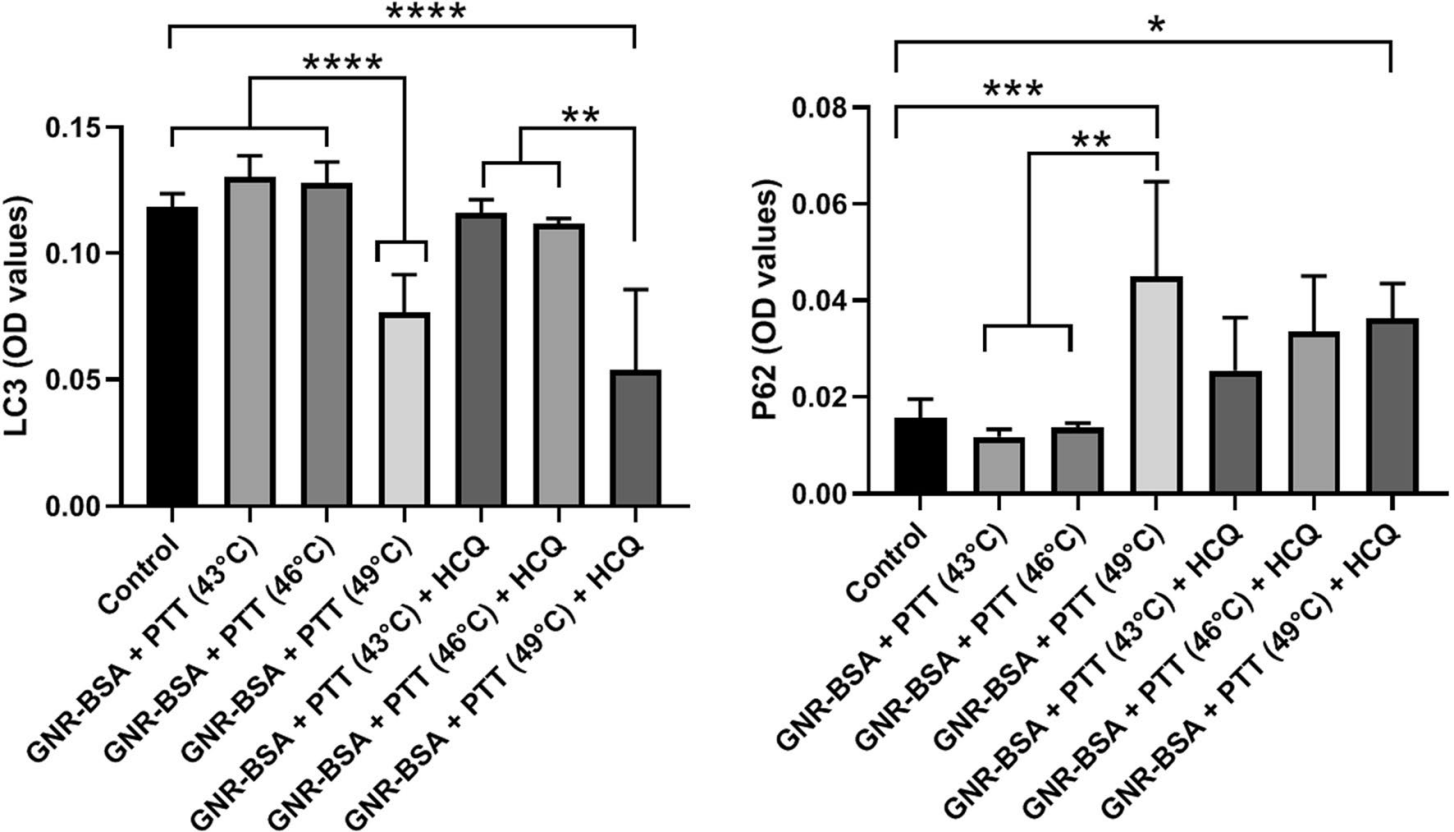
Measuring protein levels of P62 and LC3 using ELISA. Data showed the efficiency of PTT and PTT + HCQ in the alteration of both proteins related to the autophagy signaling pathway. One-Way ANOVA and Tukey post hoc analysis. *p < 0.05; **p < 0.01; ***p < 0.001 and ****p < 0.0001.

The promotion of autophagic response is touted as a basic resistance mechanism against different insulting modalities^39^. According to data obtained by ELISA, the exposure of CSCs to low and medium thermal stress (43 °C and 46 °C) induced slightly the synthesis of LC3 but these changes did not reach statistically significant differences (Fig. 5). Notably, the exposure of CSCs to 49 °C inhibited the protein levels of LC3. Likewise, the thermal stress of CSCs pre-treated with HCQ can lead to statistically significant differences compared to the control (*p* < 0.05; Fig. 5). Intracellular levels of P62/SQSTM1 are reduced upon the activation of autophagy response while the aborted autophagy leads to the accumulation of P62. This factor is involved in the elimination of ubiquitinylated particles to proteasomal degradation^40^. Therefore, it seems that the 8-min incubation of CSCs may disrupt the autophagy machinery.

### GNR-BSA and laser irradiation suppressed CSC clonogenic capacity

**Figure 6:**
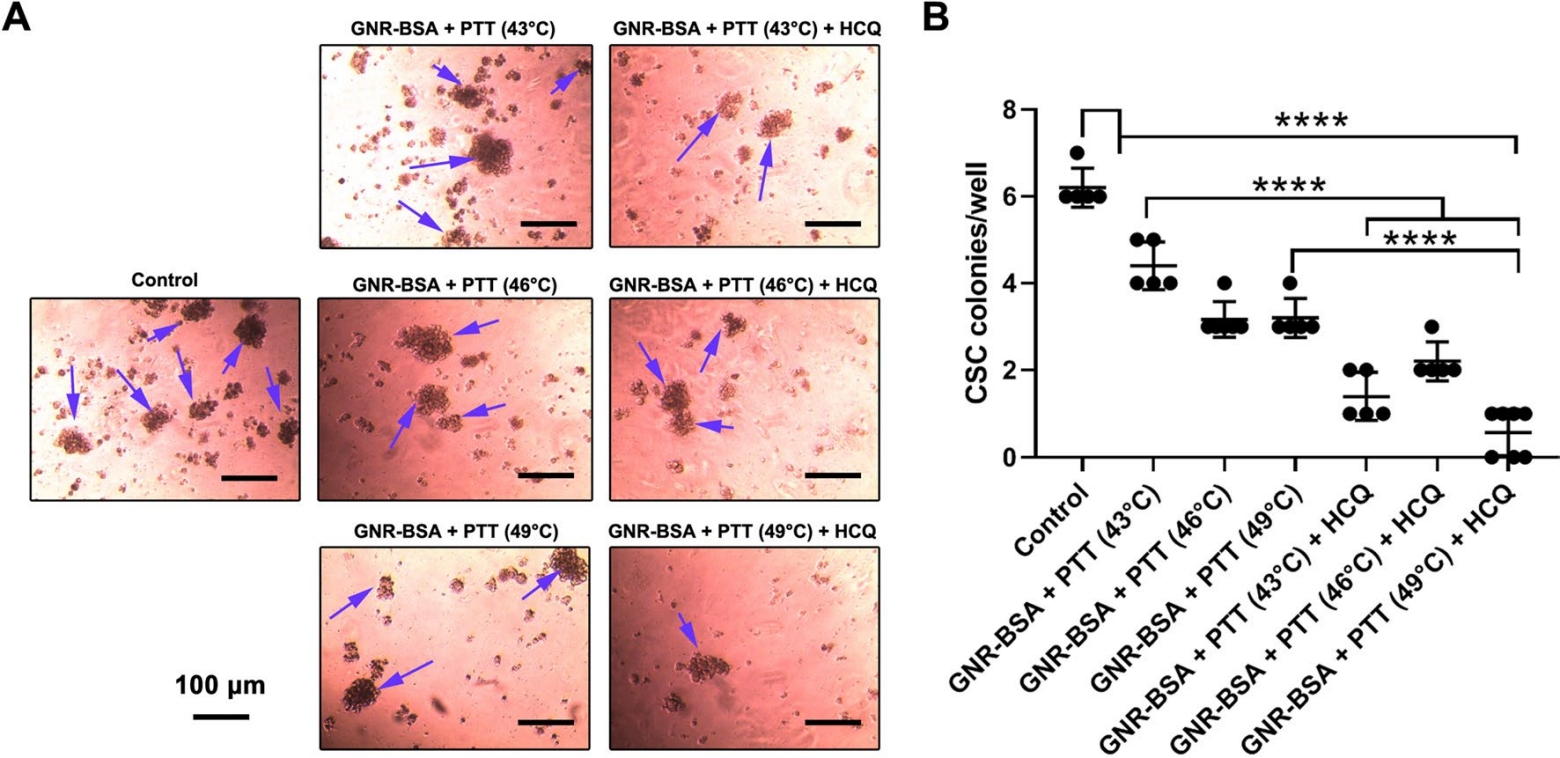
Effect of PTT and PTT + HCQ on CSC spheroid formation. Upon the increase in temperature, the clonogenic capacity of CSCs was reduced compared to the control group (blue arrows). These effects were prominent when the combination of PTT and HCQ was used. One-Way ANOVA and Tukey post hoc analysis. ****p < 0.0001.

The formation of spheroidal cell aggregates is a notable CSC feature, indicating both stemness and tumorigenic capacity^26^. On this basis, we performed a clonogenic assay to evaluate the tumoricidal effect of the PTT protocol on CD133^+^ SH-SY5Y cells. Data showed that the number of colonies was reduced 24 h after exposure to the GNR-BSA, laser irradiation, and HCQ compared to the control group (*p* < 0.05; Fig. 6). According to our data, the induction of thermal stress, 43, 46, and 49 °C in the presence and absence of HCQ led to statistically significant colony reduction compared to the control group. Noteworthy, pre-treatment of CSCs with HCQ intensified tumoricidal effects of GNR-BSA compared to groups that received PTT regime without HCQ. For instance, we found statistically significant differences in the number of colonies between GNR-BSA + PTT (43 °C) and GNR-BSA + PTT (43 °C) + HCQ, indicating the synergistic effect of HCQ with thermal therapy (Fig. 6). A similar trend was also shown CSCs exposed to 49 °C in the presence and absence of HCQ. These data demonstrated that thermal stress 43, 46, and 49 °C abrogated the clonogenic capacity of CSCs after 24 h. The addition of HCQ can increase the tumoricidal effect of thermal stress on CSCs.

## Discussion

CSCs are a small fraction of cells with stemness features that cause tumor relapse and progression^41^. Through differentiation, these cells can give rise to the different lineages during tumor propagation^41,42^. Due to inherent cell resistance against different modalities, many attempts have been collected to increase the tumoricidal effects of the current therapeutic approaches. A great body of literature has revealed the promising oncostatic effects driven by PTT in empirical studies^43^. On this basis, this study was conducted to assess the tumoricidal effects of low, medium, and high temperatures induced by GNR-BSA exposed to laser irradiation on human neuroblastoma CD133^+^ cells. GNRs have been proven to be promising agents of PTT because of their high photothermal effect and easy manipulation of optical properties to use in the near-infrared transparency windows. We synthesized and used small GNRs because the extinction of the small nanorods results mostly from absorption processes than scattering. So, the photothermal conversion efficiency is improved. CTAB-GNRs were coated with BSA for better biocompatibility and stability which was confirmed by reporting of negative zeta potential and a redshift LSPR band for GNRs after BSA coating. The synthesized GNRs-BSA exhibited a good photothermal effect for reaching to aimed temperature by the NIR laser irradiation with different intensity.

The viability of CSCs after exposure to the PTT protocol was evaluated by MTT assay which demonstrated the reduction of survival rate exposed to the combination of GNR-BSA + PTT (43, 46, and 49 °C). Here, the conjugation of BSA was done to reduce the cytotoxicity of cationic surfactant CTAB. Our data showed indicated a slight increase in the CSC survival rate in the GNR-BSA group compared to the control CSCs. Despite the slight toxicity of the GNRs, no statistically significant differences were found that correlated with remnant CTAB on the surface of GNRs^44^. Along with our data, Zhang and co-workers showed the temperature dependence of melanoma cell death folic acid-conjugated GNRs^45^. In contrast to the former study, our data showed the lack of significant differences between the groups exposed to various temperature systems in the presence or absence of HCQ. One possible reason for the lack of statistically significant differences in groups received the combination of GNR-BSA + HCQ + PTT (46 °C) and GNR-BSA + HCQ + PTT (49 °C) would be that CSCs can use different resistance mechanisms to circumvent the drug insults. For example, it is possible that CSCs can exclude HCQ in the early hours after treatment or other resistance mechanisms are provoked in HCQ-treated cells. Likewise, the pre-treatment of CSCs with HCQ did not increase PTT efficiency compared to the groups that received PTT only indicated by the MTT assay. Direct evidence for higher basal thermo-tolerance properties in CSCs compared to the normal cancer cells remain a matter of subject. The elevation of Annexin-V^+^ cells indicated by flow cytometry analysis raised the possibility of apoptosis participating in GNR-BSA-mediated PTT killing of CSCs rather than necrosis. Previously, Zhang et al. indicated the cell death pattern switching from apoptosis to necrosis concurrently by increasing the temperature from 43 to 49 °C^45^. By contrast, our results showed a slight increase in the percent of necrotic CSCs at higher temperatures induced by NIR. One reason would be that purified CD133^+^ CSCs and other CSC types possess higher resistance properties against insulting conditions related to the mass of cancer cells^46^. Simultaneous incubation of CSCs with PTT and HCQ increased the percent of CSCs entering apoptosis and especially necrosis compared to groups that received GNR-BSA plus PTT. The higher rate of apoptotic and necrotic changes in irradiated CSCs in groups pre-treated with HCQ is possibly associated with the inhibition of autophagic response, making these cells more sensitive to photothermal stress^47^. For instance, it was suggested that HCQ, as an inhibitor of autophagic efflux, can trigger certain signaling pathways like the CXCR4-CXCL12 axis, Toll-like receptor 9 signaling, and p53, leading to a reduced survival rate^48^. Although investigated to a lesser extent by previous studies, the possible effect of GNR-BSA + PTT was assessed on CSC clonogenic capacity. In the early tumorigenesis stages, the formation of spheroids is related to self-renewal capacity and malignancy^26^. Commensurate with these comments, the interruption of the spheroid formation may be a promising strategy for tumor expansion and metastasis^26^. Eight-minute incubation of neuroblastoma CSCs to low, medium, and especially higher temperatures did abort the clonogenic capacity of these cells in vitro. Indeed, the simultaneous application of PTT and HCQ represented a lower colony number compared to matched-control PTT groups. In this scenario, pre-treatment of CSCs with HCQ increased the inhibitory effect of PTT on CSC spheroid formation. It was suggested that HCQ can diminish CSCs tumorigenic and stemness properties via the suppression of specific factors such as Jak2 and DNMT1^49^. It has been shown that HCQ, as an autophagy inhibitor, can suppress the activity of BNIP3 and Beclin-1 in mesenchymal cancer cells, leading to significantly reduced spheroid formation^50^. While the incubation of these cells with an autophagy stimulator such as rapamycin allows the large-size spheroid formation and growth features^50^. Although some controversial data exist in scientific documents, autophagy participates in the stemness feature and tumorigenic capacity.

Here, we resolved to examine the protein levels of P62 and LC3 related to autophagy machinery systems using ELISA. After the exposure of CSCs to 808 nm NIR laser at different temperature conditions, protein levels of intracellular LC3 were reduced coincided with intracellular accumulation of P62 by increasing the ambient temperature from low and medium to high values. It has been shown that LC3 interacts with P62 to generate autophagosomes and autophagy inhibition can contribute to P62 accumulation^51^. Monitoring the expression of autophagy machinery components revealed up-regulation and down-regulation of factors from different signaling transduction pathways. Among the co-regulators of autophagy and apoptosis, the expression of TNF-α was readily increased by elevating the PTT temperature to 49 °C which allows autophagy-mediated apoptotic CSC death. On the other hand, an increased expression of PIK3CG (~ 266-fold) in CSCs at medium and higher temperatures can lead to the inhibition of apoptotic changes via regulating the phosphorylation of Akt^52^. Commensurate with these descriptions, the expression of Akt was significantly suppressed in higher temperatures. Increased levels of ATG9B, involved in vacuole formation, were obtained 24 h after CSCs exposure to 43, 46, and 49 °C are thought to be compensatory resistance mechanisms. However, we believed that the induction rate was blunted to some extent at higher temperatures. Besides, among the majority of genes participating in autophagic vacuole formation, the expression of ATG5, ATG7, ATG9A, and AMBRA1 displayed the lowest in groups that received 49 °C for 8 min, showing inhibition of vacuole formation in a temperature-dependent manner. It is thought that the inhibition of autophagic transfer and vacuole formation mighty direct the cells to apoptotic changes^53^. The significant expression of GABARAP showed the activation of certain pathways involved in vacuoles targeting in response to the ambient temperature of 43 and 46 °C while these effects were blunted at group 49 °C. Of co-regulators genes related to autophagy and the cell cycle, the expression of INFG was remarkably reduced in groups with 46 and 49 °C PTT, abortion of certain mechanisms related to CSCs proliferation. Similar to changes, the induction of HSP90AA1, HSPA8 indicated chaperone-mediated autophagic response and apoptosis in CSCs exposed to different PTT regimes^54^. These data indicated that the current modality can induce the formation of misfolded and denatured proteins under high-temperature conditions that predispose the CSCs to undergo the apoptotic changes by engaging unfolded-protein response machinery signaling^55^. There are some limitations in the current study and future studies are requested to address these issues. Here, we explored the combined tumoricidal effect of GNRs and laser irradiation on CD133^+^ cells (CSCs) without the application of an active targeting system. It is suggested that future studies use sophisticated systems with the specific interaction between the surface CD133 receptor and GNRs to increase the targeting delivery of GNRs in the CSC population. Application of modalities based on anti-CD133^+^ antibodies, aptamers, and peptides can yield better oncostatic outcomes^56–58^. Monitoring the expression of stemness-related markers can help us to forecast the rate of phenotype shifting in the stemness and non-stemness status. It is better to monitor the surface distribution of CD133 in CSCs over the experimental period.

## Conclusions

Compared to previous experiments, our data exhibit that irradiated GNRs can exert tumoricidal effects on CD133^+^ CSCs by the dysregulation of autophagy machinery in a temperature-dependent manner. It seems that in mild to moderate temperatures such as 43 and 46 °C, autophagic and apoptotic cell death are prominent in CSCs exposed to PTT. By contrast, higher temperatures led to suppression of autophagic response and conversion of CSC death from apoptosis to necrosis. Due to the close interaction of the autophagy signaling pathway with apoptosis, the establishment of de novo therapeutic approaches targeting both autophagic and apoptotic cell death can circumvent problems associated with rapid necrotic changes. It should not be forgotten that inadequate heating in lower temperatures can increase CSCs resistance via engaging protective autophagic mechanisms. Having a dual role in the dynamic growth of cells and crosstalk with the apoptosis pathway, the stimulation of autophagy effectors should be done carefully to trigger apoptotic changes in a controlled manner. It is also possible that several tumor cells within the tumor microenvironment like CSCs and normal cancer cells display different autophagic responses after being exposed to PTT regimes. Future studies should investigate the simultaneous photothermal activity of GNRs on CSCs and other tumor cells.

## Data Availability

All data generated or analyzed during this study are included in the published article.

## Abbreviations

MTT: 3-(4, 5-Dimethylthiazol-2-yl)-2, 5-diphenyltetrazolium bromide
BSA: Bovine serum albumin
CSCs: Cancer stem cells
DLS: Dynamic light scattering
DW: Deionized water
EDTA: Ethylenediaminetetraacetic acid
FITC: Fluorescein isothiocyanate
FBS: Fetal bovine serum
GNRs: Gold nanorods
HAuCl_4_: Hydrogen tetrachloroaurate(III) hydrate
CTAB: Cetyltrimethylammonium bromide
HCQ: Hydroxychloroquine
DMEM/LG: Low-glucose content Dulbecco’s modified eagle medium
MACS: Magnetic activated cell sorting
Pen-Strep: Penicillin-Streptomycin
PTT: Photothermal therapy
AgNO3: Silver nitrate
NaBH_4_: Sodium borohydride
SDS: Sodium dodecyl sulfate
TEM: Transmission electron microscopy
TBST: Tris-buffered saline-0.1% Tween 20
